# Epinephrine May Contribute to the Persistence of Traumatic Memories in a Post-traumatic Stress Disorder Animal Model

**DOI:** 10.3389/fnmol.2020.588802

**Published:** 2020-10-26

**Authors:** Raquel Martinho, Ana Oliveira, Gabriela Correia, Márcia Marques, Rafaela Seixas, Paula Serrão, Mónica Moreira-Rodrigues

**Affiliations:** ^1^Laboratory of General Physiology, Institute of Biomedical Sciences Abel Salazar, University of Porto (ICBAS/UP), Porto, Portugal; ^2^Center for Drug Discovery and Innovative Medicines, University of Porto (MedInUP), Porto, Portugal; ^3^Department of Pharmacology and Therapeutics, Faculty of Medicine, University of Porto (FMUP), Porto, Portugal

**Keywords:** epinephrine, norepinephrine, post-traumatic stress disorder, epinephrine deficient mice, phenylethanolamine-*N*-methyltransferase-knockout mice, hippocampus, Nr4a transcription factors

## Abstract

The importance of catecholamines in post-traumatic stress disorder (PTSD) still needs to be explored. We aimed to evaluate epinephrine’s (EPI) causal role and molecular mechanism for the persistence of PTSD traumatic memories. Wild-type (WT) and EPI-deficient mice (phenylethanolamine-*N*-methyltransferase-knockout mice, Pnmt-KO) were induced with PTSD and behavioral tests were performed. Some Pnmt-KO mice were administered with EPI or vehicle. Catecholamines were quantified by HPLC-ED. *Nr4a1*, *Nr4a2*, and *Nr4a3* mRNA expression were evaluated by real-time PCR in hippocampus samples. It was observed an increase in EPI and freezing behavior, and a decrease in open arm entries in the elevated plus-maze test and time spent in the light in the light–dark test in WT mice in the PTSD-induction group compared to control. After induction of PTSD, Pnmt-KO mice showed a decrease in freezing, as well as an increase in open arm entries and transitions between compartments compared to WT. After PTSD induction, Pnmt-KO mice administered with EPI showed an increase in freezing compared with the vehicle. On day 0 of PTSD induction, it was observed an increase in mRNA expression of *Nr4a2* and *Nr4a3* genes in the hippocampus of WT mice compared to control, contrary to Pnmt-KO mice. In conclusion, our data suggest that EPI may be involved in the persistence of traumatic memories in PTSD, possibly through enhancement of the expression of *Nr4a2* and *Nr4a3* genes in the hippocampus. Peripheral administration of EPI restored contextual traumatic memories in Pnmt-KO mice, which suggests a causal role for EPI. The persistence of contextual traumatic memories may contribute to anxiety-like behavior and resistance of traumatic memory extinction in this PTSD mice model.

## Introduction

The autonomic nervous system mobilizes the body’s resources under stress and induces the “freeze, fight or flight” response, important for survival and adaptation in nature. To maintain homeostasis, the sympathetic nervous system is constantly being activated (Lipov, 2014). However, in some circumstances, stress may cause pathology, as is the case for post-traumatic stress disorder (PTSD) and other anxiety disorders (Crawley, 1981; Crawley et al., 1984; Maercker et al., 2013).

Patients with PTSD usually have three major features: re-experience the stressful event, attempt to avoid reminders of the trauma, and a state of hyperarousal, such as increased hypervigilance and startle response (van der Kolk, 2000; Pole, 2007; Pitman et al., 2012; American Psychiatric Association, 2013; Yehuda et al., 2015). Although these symptoms are common after the traumatic event, they abnormally persist in individuals with PTSD. This disorder can thus be seen, in part, as a failure to recover from the normal reaction to the trauma (Yehuda et al., 1992). Therefore, patients with PTSD show deficits in the extinction of emotional memories and have persistent severe anxiety (Lissek et al., 2005; Inslicht et al., 2013).

Some studies have suggested that epinephrine (EPI) is an important hormone for long-term memory consolidation in human and animal subjects (Cahill and Alkire, 2003; Dornelles et al., 2007). Moreover, we also have shown in previous studies that mice deficient in EPI (phenylethanolamine-*N*-methyltransferase-knockout, Pnmt-KO mice) have reduced contextual fear learning (Toth et al., 2013; Alves et al., 2016). It has been documented in PTSD patients an increase of stress hormones in urine, namely EPI and norepinephrine (NE; Yehuda et al., 1992; Lemieux and Coe, 1995; Pitman and Delahanty, 2005). The increase of stress hormones can facilitate the strengthening of traumatic memories (Pitman, 1989; Pitman and Delahanty, 2005), but the action mechanism of these catecholamines in PTSD mice models needs to be explored.

Several animal models have been developed using different types of traumatic events to study PTSD in humans (Pynoos et al., 1996). The PTSD animal model that we have selected is centered on the concept that exposure to foot shocks displays the pathophysiological process and the core symptomatology of PTSD, including freezing and anxiety-like behavior (Li et al., 2006; Zhang et al., 2012; Verma et al., 2016).

*Nr4a* is a subfamily of orphan nuclear receptors genes. These genes encode for three transcription factors, namely *Nr4a1*, *Nr4a2*, and *Nr4a3*, which mediate several cellular responses (Hazel et al., 1988; Law et al., 1992; Ohkura et al., 1996). Besides their involvement in physiologic processes such as energy metabolism, inflammatory responses, and hypothalamic-pituitary-adrenal axis regulation (Wilson et al., 1993; Honkaniemi et al., 1994; Murphy and Conneely, 1997; Maira et al., 1999), these genes have been implicated in contextual fear memory formation (Liu et al., 1994; Woronicz et al., 1994; Fernandez et al., 2000; Pei et al., 2006; Rojas et al., 2007; Hawk and Abel, 2011; Oliveira et al., 2018). Previous studies showed that contextual fear conditioning induces *Nr4a* genes transcription in the hippocampus, a brain structure involved in the acquisition of contextual fear conditioning in response to stress stimuli (von Hertzen and Giese, 2005; Hawk et al., 2012; Mizuno et al., 2012; Oliveira et al., 2018). Thus, the study of *Nr4a* genes expression in this model may be relevant. The present study aimed to understand the influence of EPI in the persistence of PTSD traumatic memories and possible molecular mechanisms.

## Materials and Methods

### Animals

All animal care and experimental protocols were carried out following European Directive number 2010/63/EU, transposed to Portuguese legislation by Directive Law 113/2013 and 1/2019, and approved by the Organism Responsible for Animal Welfare in Faculty of Medicine of University of Porto and National Authority for Animal Health. The Pnmt-KO mice (Pnmt^−/−^) were produced by the insertion of the Cre-recombinase gene into the locus encoding for the Pnmt enzyme, which results in EPI’s deficiency in homozygous (Pnmt^−/−^). Pnmt-KO mice were first generated in C57BL/6 mice (Ebert et al., 2004) and these mice were fully bred in 129x1/SvJ background. Steven N. Ebert kindly provided a couple of Pnmt-KO (Pnmt^−/−^) and heterozygous (Pnmt^+/−^) mice and they were bred in our conventional vivarium. Regarding breeding, 1 male and 2 females of heterozygous (Pnmt^+/−^) mice siblings were placed together for mating. Pups were weaned 21 days after birth, males and females were separated, and placed in individual cages. Genotypes at the Pnmt locus were identified by polymerase chain reaction (PCR) of ear DNA, as described before (Ebert et al., 2004). From heterozygous couples (Pnmt^+/−^) we selected wild-type (WT) female and WT male mice (Pnmt^+/+^) or Pnmt-KO female and Pnmt-KO male mice (Pnmt^−/−^), which were siblings, and were placed in the same cage to breed WT (Pnmt^+/+^) or Pnmt-KO (Pnmt^−/−^) mice, respectively. WT (*n* = 45) and Pnmt-KO (*n* = 41) female mice (8–12 weeks old) were kept under controlled environmental conditions (12 h light/dark cycle, room temperature 23 ± 1°C, humidity 50%, autoclaved drinking water, mice diet 4RF25/I and 4RF21/A; Mucedola, Porto, Portugal) in the same room and housed with the respective litter. When female rodents are placed in small groups and share a recirculated air supply it is observed a synchrony of their ovarian cycles, which is mediated by pheromones (McClintock, 1978; Schank and McClintock, 1992). During the 5 days of habituation before experiments, all mice were handled once daily with gloved hands for 2 min. Also, all experimental groups in a protocol were performed on the same day, not more than 2 h apart. Therefore, the step of measuring the estrous cycle for each individual was waived.

### PTSD Mice Model

**Figure 1:**
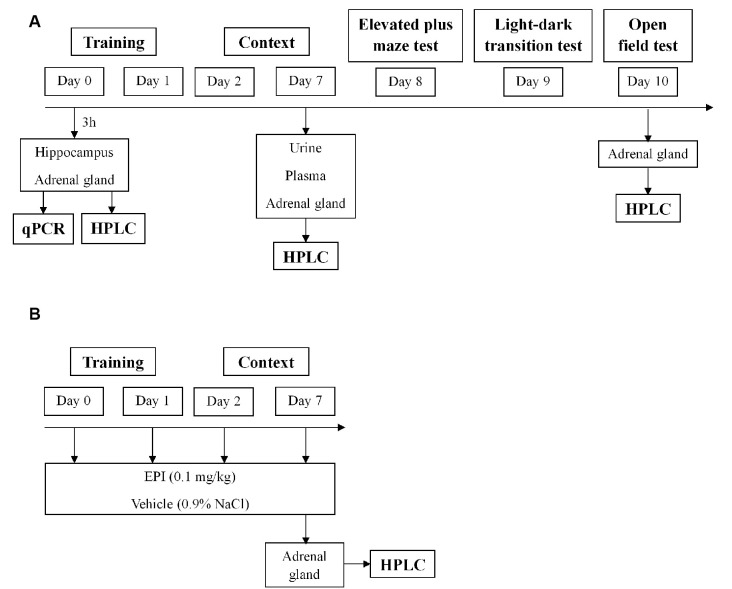
Schematic representation of behavioral protocols, experimental design, treatments, and sample collection. **(A)** Results in Figures 2–7, 9; **(B)** results in Figure 8. HPLC, high-performance liquid chromatography; qPCR, real-time polymerase chain reaction.

As previously described (Li et al., 2006; Zhang et al., 2012; Verma et al., 2016), mice were exposed to an aversive procedure consisting of two training sessions (day 0 and day 1). For the training sessions, we used a clear Plexiglass chamber with a metal grid floor, wired to a stimulus generator. On these days, the mice had a 2-min habituation period and then they were then submitted to 15 electric shocks (intensity, 0.8 mA; duration, 10 s; the interval between sessions, 10 s), during a total time of 5 min. After training, the mice were re-exposed on days 2 and 7 to the aversive context. Re-exposure consisted of introducing the mice in the same chamber, without applying foot shocks, for 5 min. Control mice were placed in the same chamber and during the same amount of time and days as the other mice (days 0, 1, 2, and 7), but did not receive the electric shocks. The mice’s behavior was recorded with a digital video camera Sony HDR-CX405 (Sony Corporation, Japan). Freezing was defined as the absence of movement except for respiration for at least 3 s (Valentinuzzi et al., 1998). The percentage of accumulated freezing time was then calculated. Vocalization response was defined as the audible vocalization and jump response was defined as the removal of at least three paws from the grid floor in response to the shock (Rocinholi et al., 1997). All quantifications were performed manually and blinded. In the first protocol, WT mice were induced with PTSD (PTSD-induction group) or not (control group) until day 7. Then, urine, plasma, and adrenal gland were collected for catecholamines evaluation by HPLC-ED (Figure 1A). In the second protocol, freezing behavior was evaluated in WT and Pnmt-KO mice (PTSD-induction group or control group) on days 2 and 7. Afterward, elevated plus maze (day 8), light–dark transition (day 9), and open field (day 10) tests were conducted (Figure 1A). At the end of protocols mice were anesthetized (ketamine, 100 mg/kg and xylazine, 10 mg/kg; i.p.), and blood and left adrenal glands were collected (Figure 1A). In the third protocol, EPI (0.1 mg/kg, i.p., 3 min; Lee et al., 2001) or vehicle (0.9% NaCl) were administered to Pnmt-KO mice (PTSD-induction group) on days 0, 1, 2, and 7 and freezing behavior was evaluated (Figure 1B). In the fourth protocol, the same procedure was repeated just on day 0 with WT and Pnmt-KO mice (PTSD-induction group or control group) and the hippocampus was collected for real-time PCR (qPCR; Figure 1A).

### Metabolic Studies

WT mice were placed in metabolic cages (Tecniplast, Buguggiate-VA, Italy) for 24-h urine collection on day 7 for later determination of catecholamines, as previously described (Moreira-Rodrigues et al., 2007b, 2010, 2012). Twenty-four hours food and water intake and feces weight were evaluated. The urine vials for catecholamines quantification contained 60 μl of hydrochloric acid (6 mol/l) to avoid spontaneous oxidation of the amines and its derivatives. All urine samples were frozen at −80°C until further use.

### Quantification of Catecholamines

The left adrenal gland was collected and emerged in perchloric acid 0.2 M overnight, at 4°C, and kept under −80°C until further use. Afterward, the adrenal glands supernatant was centrifuged for 2 min (2,700× *g* and 4°C) and diluted. Blood was also collected by left ventricle puncture to a heparinized tube, then the samples were centrifuged (1,000× *g*, 15 min, 4°C) and kept at −80°C until further use. The catecholamines present in plasma and urine were concentrated by the alumina method, as previously described (Moreira-Rodrigues et al., 2007b, 2010, 2012, 2014). Catecholamines in the adrenal gland, plasma, and urine samples were separated by reverse-phase high-performance liquid chromatography (HPLC) and quantified by electrochemical detection. The detection limit was between 350 fmol and 1,000 fmol.

### Other Behavioral Testing

#### Elevated Plus Maze Test

Eight days after PTSD induction, an elevated plus-maze test was conducted as previously described (Pellow et al., 1985; Popovitz et al., 2019). Briefly, the apparatus consisted of two open arms (40 × 10 cm) alternating at right angles with two arms enclosed by 20 cm high walls. The four arms delimited a central area of 5 cm^2^. The whole apparatus was placed 60 cm above the floor. The test began by placing the animal in the center with its head facing a closed arm. The visits in open and closed arms and total arm entries for 5 min were recorded with a digital video camera Sony HDR-CX405 (Sony Corporation, Japan), analyzed manually and blinded, and a four paws criterion was used for arm entries (Li et al., 2006).

#### Light–Dark Transition Test

Nine days after PTSD induction, the light–dark transition test was carried out as previously described (Li et al., 2006). The apparatus consisted of a wooden chamber subdivided into a dark (50 × 20 × 25 cm) and a light compartment (50 × 30 × 25 cm). The compartments were connected by a small divider (10 × 10 cm). Each animal was placed into the light compartment facing the opposite wall to the entry. The latency of the first entry into the dark compartment, the time spent in each compartment, and the number of transitions between compartments were recorded with a digital video camera Sony HDR-CX405 (Sony Corporation, Japan) for 5 min and analyzed manually and blinded.

#### Open Field Test

Ten days after PTSD induction, the open field test was conducted as described before (Zhang et al., 2012; Inoue et al., 2018). The open field wooden chamber (50 × 50 × 30 cm) had black lines on the floor delineating twelve peripheral squares (12.5 × 12.5 cm) and a central square (25 × 25 cm). Each animal was placed in the corner of the arena and locomotor activity was recorded for 10 min with a digital video camera Sony HDR-CX405 (Sony Corporation, Japan). The total distance traveled was analyzed using ToxTrac ver 2.84 (open source software freely available at sourceforge.net/projects/toxtrac, Rodriguez et al., 2017, 2018; Henry et al., 2019). The number of squares crossed, entries in the center, and feces were analyzed manually and blinded.

### RNA Isolation and Relative Quantification of mRNA Expression

qPCR was performed in hippocampus samples collected 3 h after day 0 of PTSD induction, as previously described (Moreira-Rodrigues et al., 2007a; Mendes et al., 2018; Oliveira et al., 2018). Total RNA isolation was carried out with the illustra^TM^ RNAspin Mini RNA Isolation Kit (GE Healthcare Life Sciences, Buckinghamshire, UK). The membrane desalting step of the kit created a chemical environment for efficient on-column DNase I digest and thus the isolated total RNA was free of genomic DNA. The concentration and purity of the isolated RNA were measured using the NanoDrop 2000 spectrophotometer (Thermo Fisher Scientific, Waltham, MA, USA). Reverse transcription was performed in a T100^TM^ Thermal Cycler (Bio-Rad, Hercules, CA, USA) using a Reverse Transcription kit (NZY First-Strand cDNA Synthesis Kit NZYTech—Genes and Enzymes, Lisbon, Portugal). qPCR reactions were carried out in StepOne^TM^ real-time PCR System (Applied BioSystems, Waltham, MA, USA). Gene-specific primers (10 μM), Maxima SYBR Green qPCR Master Mix (Thermo Fisher Scientific, Waltham, MA, USA), and Nuclease-free H_2_O (Thermo Fisher Scientific, Waltham, MA, USA) were mixed and cDNA was added (1:20). As negative controls, a no template control and a minus reverse transcriptase control were used. The cycling parameters were as follows: denaturation at 95°C for 30 s, annealing at 60°C for 1 min, and extension at 60°C for 1 min (40 cycles). Gene-specific primers and the size of amplified DNA fragments are in Table 1. Results of mRNA quantification are expressed in an arbitrary unit (AU) after normalization for Glyceraldehyde 3-phosphate dehydrogenase (GAPDH).

**Table 1:** Primers used in gene expression analysis.

Gene	Primer (5′ 3′)	Size (bp)
*Nr4a1*	F: AAAATCCCTGGCTTCATTGAG	102
	R: TTTAGATCGGTATGCCAGGCG	
*Nr4a2*	F: CGGTTTCAGAAGTGCCTAGC	214
	R: TTGCCTGGAACCTGGAATAG	
*Nr4a3*	F: GTGGCTCGACTCCATTAAAGAC	144
	R: GTGCATAGCTCCTCCACTCTCT	
*Gapdh*	F: CCATCACCATCTTCCAGGAG	322
	R: GCATGGACTGTGGTCATGAG	

*Nr4, Nuclear receptor 4; Gapdh, Glyceraldehyde 3-phosphate dehydrogenase; F, forward primer; and R, reverse primer*.

### Drugs

(-)-epinephrine (EPI) and perchloric acid were purchased from Sigma–Aldrich (St. Louis, MO, USA). Ketamine (Imalgene 1000, Merial, Lisbon, Portugal) and xylazine (Rompum 2%, Bayer, Lisbon, Portugal) were purchased from Agrofauna (Vila Nova de Gaia, Portugal).

### Statistics

**Figure 2:**
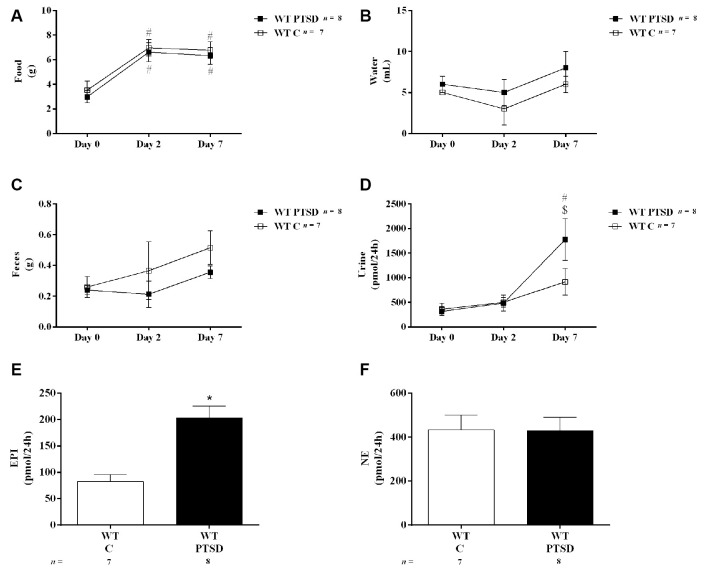
Twenty-four hours **(A)** food and **(B)** water intake, **(C)** feces weight, **(D)** urine volume, and urinary **(E)** epinephrine (EPI) and **(F)** norepinephrine (NE) quantity in 24 h of wild-type (WT) mice in the post-traumatic stress disorder (PTSD) induction group or control group (WT C) on day 7 of PTSD induction. Values are means ± standard error of the means (SEM) of 7–8 mice per group. WT C, WT control mice, placed in the same chamber during the same amount of time and days as the other mice but were not induced with PTSD; WT PTSD, WT mice in the PTSD-induction group. ^#^Significantly different from correspondent values on day 0 of respective group (*p* < 0.05). ^$^Significantly different from correspondent values on day 2 of the respective group (*p* < 0.05). *Significantly different from correspondent values in WT control mice (*p* < 0.05).

**Figure 3:**
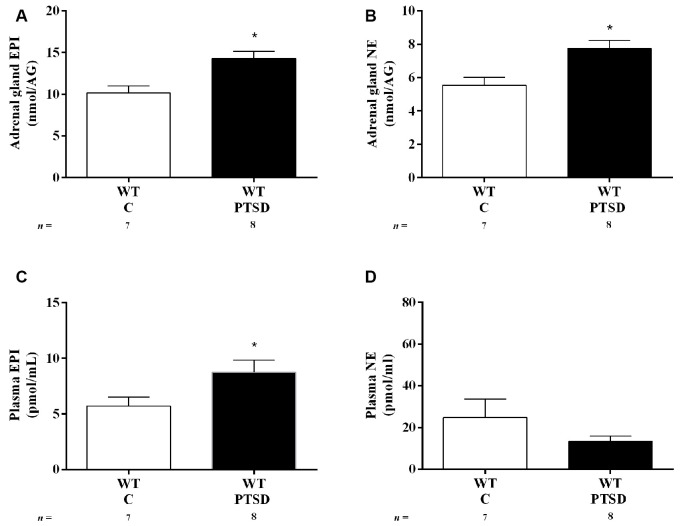
Concentration of **(A,C)** epinephrine (EPI) and **(B,D)** norepinephrine (NE) in **(A,B)** adrenal gland and **(C,D)** plasma of WT mice in the PTSD induction group or control group (WT C) on day 7 of PTSD induction. Values are means ± SEM of 7–8 mice per group. WT C, WT control mice, placed in the same chamber during the same amount of time and days as the other mice but were not induced with PTSD; WT PTSD, WT mice in the PTSD-induction group. *Significantly different from correspondent values in WT control mice (*p* < 0.05).

**Figure 4:**
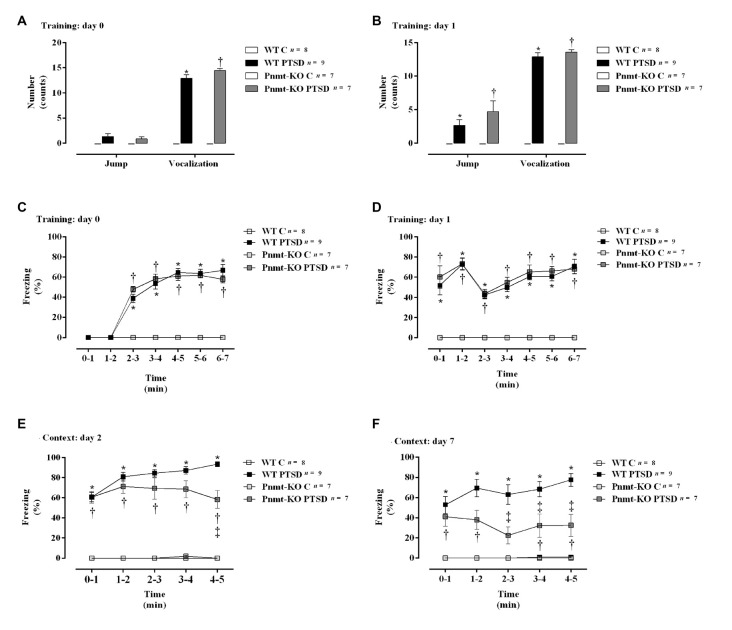
**(A,B)** Shock responsivity on training sessions (days 0 and 1) and **(C–F)** freezing behavior during induction of PTSD. Freezing on **(C)** day 0, **(D)** day 1, **(E)** day 2, and **(F)** day 7 in WT and phenylethanolamine-*N*-methyltransferase-knockout (Pnmt-KO) mice in the PTSD-induction group or not (control, WT C and Pnmt-KO C). Values are means ± SEM of 7–9 mice per group. C, control mice, placed in the same chamber during the same amount of time and days as the other mice but were not induced with PTSD; WT C, WT control mice; WT PTSD, WT mice in the PTSD-induction group; Pnmt-KO C, Pnmt-KO control mice; Pnmt-KO PTSD, Pnmt-KO mice in the PTSD-induction group. *Significantly different from correspondent values in WT control mice (*p* < 0.05). ^†^Significantly different from correspondent values in Pnmt-KO control mice (*p* < 0.05). ^‡^Significantly different from correspondent values in WT mice with similar pathology (*p* < 0.05).

**Figure 5:**
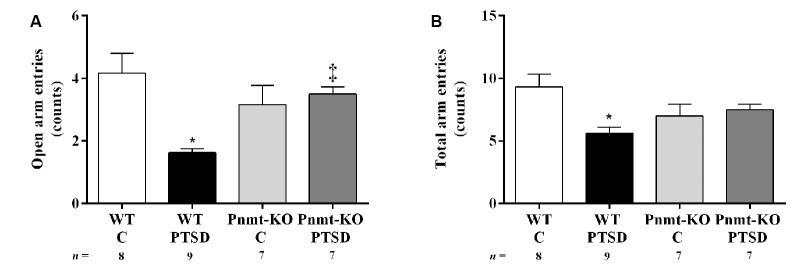
Elevated-plus maze test results in WT and phenylethanolamine-*N*-methyltransferase-knockout (Pnmt-KO) mice in the PTSD induction group or control group (WT C and Pnmt-KO C) 8 days after PTSD induction. The number of **(A)** open arm entries and **(B)** total arm entries. Values are means ± SEM of 7–9 mice per group. C, control mice, placed in the same chamber during the same amount of time and days as the other mice but were not induced with PTSD; WT C, WT control mice; WT PTSD, WT mice in the PTSD-induction group; Pnmt-KO C, Pnmt-KO control mice; Pnmt-KO PTSD, Pnmt-KO mice in the PTSD-induction group. *Significantly different from correspondent values in WT control mice (*p* < 0.05). ^‡^Significantly different from correspondent values in WT mice with similar pathology (*p* < 0.05).

**Figure 6:**
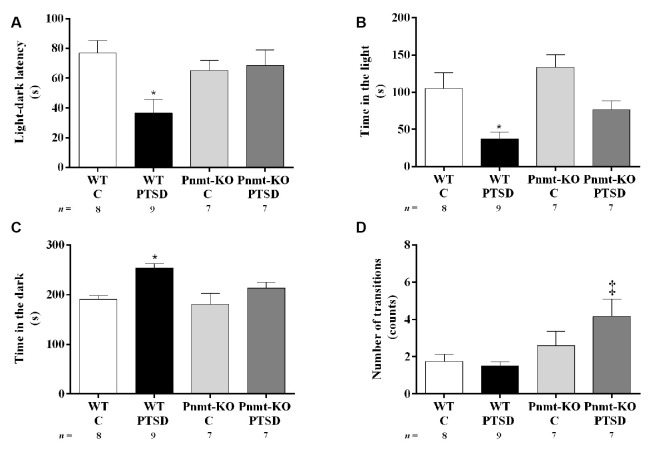
Light–dark transition test results in WT and phenylethanolamine-*N*-methyltransferase-knockout (Pnmt-KO) mice in the PTSD induction group or control group (WT C and Pnmt-KO C) 9 days after PTSD induction. **(A)** Latency to enter the dark compartment, time spent in the **(B)** dark, **(C)** light compartment, and **(D)** the total number of transitions between compartments. Values are means ± SEM of 7–9 mice per group. C, control mice, placed in the same chamber during the same amount of time and days as the other mice but were not induced with PTSD; WT C, WT control mice; WT PTSD, WT mice in the PTSD-induction group; Pnmt-KO C, Pnmt-KO control mice; Pnmt-KO PTSD, Pnmt-KO mice in the PTSD-induction group. *Significantly different from correspondent values in WT control mice (*p* < 0.05). ^‡^Significantly different from correspondent values in WT mice with similar pathology (*p* < 0.05).

**Figure 7:**
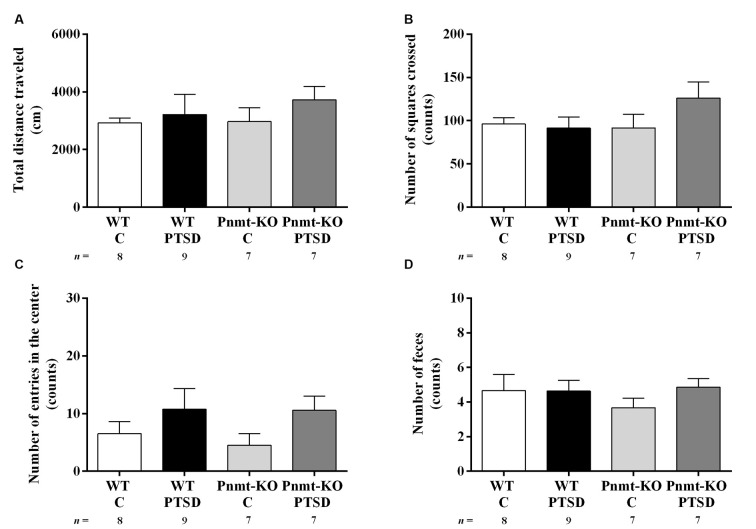
Open field test results in WT and phenylethanolamine-*N*-methyltransferase-knockout (Pnmt-KO) mice in the PTSD induction group or control group (WT C and Pnmt-KO C) 10 days after PTSD induction. **(A)** Total distance traveled and the number of **(B)** squares crossed, **(C)** entries in the center, and **(D)** feces. Values are means ± SEM of 7–9 mice per group. C, control mice, placed in the same chamber during the same amount of time and days as the other mice but were not induced with PTSD; WT C, WT control mice; WT PTSD, WT mice in the PTSD-induction group; Pnmt-KO C, Pnmt-KO control mice; Pnmt-KO PTSD, Pnmt-KO mice in the PTSD-induction group.

**Figure 8:**
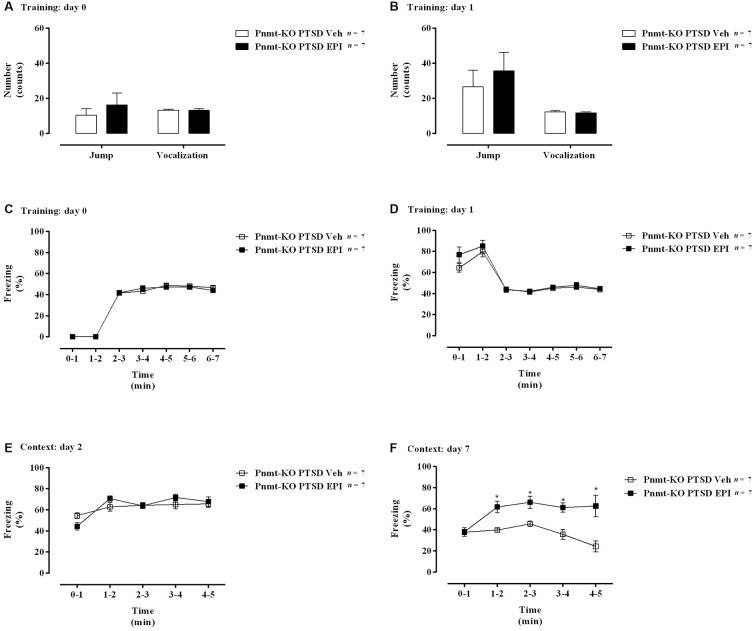
**(A,B)** Shock responsivity on training sessions and **(C–F)** freezing behavior during the induction of PTSD. Freezing on **(C)** day 0, **(D)** day 1, **(E)** day 2, and **(F)** day 7 in phenylethanolamine-*N*-methyltransferase-knockout (Pnmt-KO) mice in the PTSD-induction group administered with epinephrine (EPI) or vehicle (Veh). Values are means ± SEM of 7 mice per group. Pnmt KO PTSD EPI, Pnmt-KO mice in the PTSD-induction group administered with epinephrine. Pnmt-KO PTSD Veh, Pnmt-KO mice in the PTSD-induction group administered with the vehicle. *Significantly different from correspondent values in Pnmt-KO mice in the PTSD-induction group administered with vehicle (*p* < 0.05).

**Figure 9:**
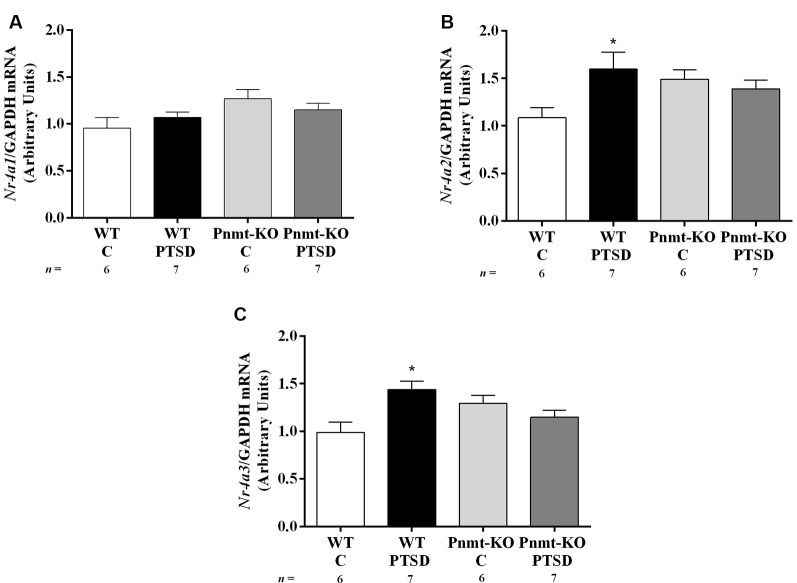
Hippocampus mRNA expression of **(A)** *Nr4a1*, **(B)** *Nr4a2*, and **(C)** *Nr4a3* in WT and phenylethanolamine-*N*-methyltransferase-knockout (Pnmt-KO) mice in the PTSD induction group or control group (WT C and Pnmt-KO C) after day 0 of PTSD induction. Values are means ± SEM of 6–7 mice per group. Results of mRNA are expressed as arbitrary units (AUs) after normalization for Glyceraldehyde 3-phosphate dehydrogenase (GAPDH). C, control mice, placed in the same chamber during the same amount of time and days as the other mice but were not induced with PTSD; WT C, WT control mice; WT PTSD, WT mice in the PTSD-induction group; Pnmt-KO C, Pnmt-KO control mice; Pnmt-KO PTSD, Pnmt-KO mice in the PTSD-induction group. *Significantly different from correspondent values in WT control mice (*p* < 0.05).

All results were presented as means ± standard error of the means (SEM). For parameters measured during metabolic studies (two groups; WT mice in the PTSD-induction group and WT control mice; Figure 2). Two-way analysis of variance (ANOVA) repeated measures followed by Tukey’s *post hoc* test was performed using pathology as “between-subjects factor” and time as “within-subjects factor” (repeated measure). For the catecholamines concentration results (two groups; WT mice in the PTSD-induction group and WT control mice; Figures 2, 3) an unpaired *t*-test was used to determine group differences. For vocalization and jump responses (four groups; WT and Pnmt-KO in the PTSD groups or controls; Figure 4). Two-way ANOVA followed by Tukey’s *post hoc* test was performed where the main effect of genotype, pathology, and genotype × pathology interaction was evaluated. Also, for vocalization and jump responses (two groups; Pnmt-KO in the PTSD group administered with EPI or vehicle; Figure 8), an unpaired *t*-test was used to determine group differences. For freezing behavior (four groups; WT and Pnmt-KO in the PTSD groups or controls; Figure 4). Three-way ANOVA repeated measures followed by Tukey’s *post hoc* test was performed using genotype and pathology as “between-subjects factor,” and time as “within-subjects factor” (repeated measure). Also, for freezing behavior (two groups; Pnmt-KO in the PTSD group administered with EPI or vehicle; Figure 8). Two-way ANOVA repeated measures followed by Sidak’s *post hoc* test was performed using treatment as “between-subjects factor” and time as “within-subjects factor” (repeated measure). Furthermore, for elevated plus maze test, light–dark transition test, open field test, and qPCR results (four groups; WT and Pnmt-KO in the PTSD groups or controls; Figures 5–7, 9). Two-way ANOVA followed by Tukey’s *post hoc* test was performed and the main effect of genotype, pathology, and genotype × pathology interaction was evaluated. Effect sizes were estimated by calculating Cohen’s *d* test for pairwise comparisons and partial eta squared ($\eta_{\text{p}}^{2}$) for ANOVA. The correlations were evaluated by calculating Pearson’s correlation coefficient *r*. The statistically significant difference was set at *p* < 0.05.

## Results

### EPI Increases After PTSD Induction

To evaluate catecholamines levels in this PTSD mice model, WT mice were put in metabolic cages. In both WT mice in the PTSD-induction and control groups, food intake was significantly increased in both days 2 and 7 when compared to day 0 (Figure 2A). However, no statistically significant differences were observed between these groups in food intake (Figure 2A) during the 7 days of PTSD induction. Also, no significant differences were observed in water intake (Figure 2B) and feces weight (Figure 2C) between groups. Also, 24-h urine volume was significantly increased in WT mice in the PTSD-induction group on day 7 when compared to day 2 and day 0, which was not observed in WT control mice (Figure 2D). However, no statistically significant differences in 24-h urine volume were observed between these groups of mice. Furthermore, EPI in 24-h urine was significantly increased in WT mice 7 days after induction of PTSD when compared to WT control mice (Figure 2E). Also, there was a statistically significant increase of EPI (Figure 3A) and NE (Figure 3B) in the adrenal gland, and of EPI in plasma (Figure 3C) of WT mice in the PTSD-induction group when compared to WT control mice. Moreover, significantly strong positive correlations were observed between EPI in adrenal gland and urine (*r* = 0.6615, *p* < 0.05), and between EPI in plasma and urine (*r* = 0.5076, *p* < 0.05). On the other hand, no statistically significant differences were observed in NE in urine (Figure 2F) and plasma (Figure 3D) between these groups. EPI was vestigial in the adrenal glands of Pnmt-KO mice. Details of statistical data are in Supplementary Data 1.1.

### EPI-Deficient Mice Have Less Freezing After PTSD Induction Than WT

On days 0 and 1 of the PTSD induction model, it was observed a significant increase in vocalization response in WT mice in the PTSD-induction group compared to WT control (Figures 4A,B). Moreover, no statistically significant differences in shock responsivity (vocalization and jump; Figures 4A,B) were observed between WT and Pnmt-KO mice in the PTSD-induction groups. Also, it was observed a significant increase in freezing behavior during training days 0 and 1 in WT mice in the PTSD-induction group compared to control (Figures 4C,D). No statistically significant differences in freezing behavior were observed between WT and Pnmt-KO mice PTSD-induction groups during training days 0 and 1 (Figures 4C,D).

Furthermore, after re-exposure to the aversive context, it was observed a higher freezing response in WT mice in the PTSD-induction group compared to control, on days 2 and 7 (Figures 4E,F). Moreover, significantly strong positive correlations were observed between contextual freezing on day 7 and EPI in adrenal gland (*r* = 0.4959, *p* < 0.05), plasma (*r* = 0.4001, *p* < 0.05), and urine (*r* = 0.5825, *p* < 0.05). Also, Pnmt-KO mice in the PTSD-induction group showed a significant decrease in freezing behavior compared to WT mice PTSD-induction group on days 2 and 7 (Figures 4E,F). Details of statistical data are in Supplementary Data 1.2.

### EPI-Deficient Mice Have Less Anxiety After PTSD Induction Than WT

In the elevated plus-maze test (day 8), the open arms entries (Figure 5A) and the total number of arm entries (Figure 5B) were significantly decreased in WT mice in the PTSD-induction group when compared to WT control. Also, the open arms entries were significantly increased in Pnmt-KO mice when compared to WT mice in the PTSD-induction groups (Figure 5A). In WT mice in the PTSD-induction group, significantly strong negative correlations were observed between contextual freezing on day 7 and open arms entries (*r* = −0.6263, *p* < 0.05), and total number of arm entries (*r* = −0.6440, *p* < 0.05). In other groups, no correlations were observed (*p* > 0.05).

In the light–dark transition test (day 9), WT mice in the PTSD-induction group exhibited a significant decrease in the latency to escape from the light to the dark compartment (Figure 6A) and in the time spent in the light compartment (Figure 6B) compared to WT control, and also a significant increase in the time spent in the dark compartment in this group (Figure 6C). No significant differences were observed in these parameters in Pnmt-KO mice. Also, it was observed a significant increase in the total number of transitions between compartments in Pnmt-KO mice when compared to WT mice in the PTSD-induction groups (Figure 6D). Details of statistical data are in Supplementary Data 1.3.

### PTSD Induction Did Not Affect Spontaneous Locomotor Activity

In the open field test (day 10) no statistically significant differences were observed in the total distance traveled (Figure 7A), number of squares crossed (Figure 7B), entries in the center (Figure 7C), and feces (Figure 7D) between groups. Also, in all groups, no correlations were observed between contextual freezing on day 7 and open-field parameters.

### Peripheral EPI may be Involved in the Persistence of Traumatic Memories in PTSD

On days 0 and 1 of PTSD induction model, no statistically significant differences were observed in vocalization and jump responses, and in freezing behavior between Pnmt-KO mice administered with EPI or vehicle in the PTSD-induction groups (Figures 8A–D). Also, it was observed a significant increase in freezing behavior in Pnmt-KO mice administered with EPI after 7 days of PTSD induction compared to Pnmt-KO mice administered with vehicle (Figures 8E,F). Details of statistical data are in Supplementary Data 1.4.

### EPI Appears to Contribute for the Persistence of Traumatic Memories in PTSD by Influencing Nr4a Genes Expression in the Hippocampus

It was observed a significant increase in mRNA expression of *Nr4a2* and *Nr4a3* genes in the hippocampus in the WT mice PTSD-induction group compared to WT control, which was not observed in Pnmt-KO mice. No significant differences were observed between WT and Pnmt-KO control mice regarding mRNA expression of *Nr4a* family genes. Details of statistical data are in Supplementary Data 1.5.

## Discussion

PTSD is one of the most common anxiety disorders that can develop after exposure to exceptionally horrifying or threatening events. However, the propensity to develop anxiety disorders also depends on genetic and environmental factors (Sartori et al., 2011). Women have 2 to 3 times higher risk of developing PTSD compared to men. Also, the lifetime prevalence of PTSD is about 10–12% in women and 5–6% in men (Tolin and Foa, 2006; Christiansen and Hansen, 2015; Olff, 2017). Therefore, in this study, we decided to use female mice.

Reconsolidation is capable of continuously alter the strength of memories during our lifetime (Lee, 2008) and may be regulated by adrenergic activity (Przybyslawski et al., 1999). PTSD-related memory is a maladaptive fear memory, which is decontextualized and leads to an intrusive recollection of the trauma in safe environments (Foa et al., 1989; Desmedt et al., 2015). Thus, we decided to apply a validated PTSD mice model (Li et al., 2006; Zhang et al., 2012; Verma et al., 2016) to study the influence of EPI in PTSD.

Animals experiencing a traumatic event, such as multiple foot shocks, rapidly form a strong phobia when a mild aversive experience occurs (Fanselow et al., 1993). This phenomenon may correspond to the emergence of phobias in PTSD patients (Rau et al., 2005). The administration of multiple foot shocks in this PTSD model has been confirmed to mimic the traumatic event. Moreover, contextual reminders in this PTSD animal model parallel the exposure to contextual cues present throughout an aversive stressful situation (Li et al., 2006; Zhang et al., 2012; Verma et al., 2016). This may perhaps induce the re-experiencing of the traumatic event (Gisquet-Verrier et al., 2004), which seems to be analogous to the behavioral changes seen in PTSD patients. Based on previous studies, the freezing response upon re-exposure to the shock context in this model is a measure of conditioned associative fear memory, reflecting the response to trauma-related cues as a symptom of PTSD (Siegmund and Wotjak, 2007).

Recent studies have established a relationship between stress hormones and PTSD (Shalev et al., 2008; Porhomayon et al., 2014). It has been documented in PTSD patients an increase of stress hormones, namely catecholamines, such as dopamine, NE, and EPI in urine (Yehuda et al., 1992; Lemieux and Coe, 1995). In accordance, EPI was increased in the adrenal gland, plasma, and urine of WT mice in the PTSD-induction group when compared to control and significantly strong positive correlations were observed between EPI in adrenal gland and urine, and between EPI in plasma and urine. No differences were observed in the food and water intake between WT mice in the PTSD-induction group and control. Therefore, the metabolic activity of these animals was not different and thus, tyrosine (precursor of NE and EPI in the catecholamine biosynthesis pathway) ingestion, which may come from the diet (Wurtman and Fernstrom, 1975; Aquilani et al., 2003), did not seem altered. Also, NE in the adrenal gland was increased in WT mice in the PTSD-induction group compared to control, which is the precursor of EPI. Consequently, the observed increase in urine EPI appears to be due to the raise of this catecholamine production in WT mice in the PTSD-induction group. On the other hand, EPI in Pnmt-KO mice was vestigial in the adrenal gland because these animals do not produce EPI, as previously shown (Ebert et al., 2004; Moreira-Rodrigues et al., 2014; Alves et al., 2016).

In the PTSD mice model used, WT mice showed an elevated freezing response and avoidance to the context associated with the aversive foot shock indicating that the aversive foot shocks followed by repeated reminders are a consistent and long-lasting animal model for PTSD, as previously shown (Li et al., 2006; Zhang et al., 2012). The absence of differences in freezing, vocalization, and jump responses in both training days (days 0 and 1) between WT and Pnmt-KO mice in the PTSD-induction group suggests that pain perception of the foot shock and acquisition of contextual fear memories were not affected by genotype (absence of EPI). Besides, pain perception and acquisition were also not affected by EPI administration.

We have previously shown that EPI strengthens contextual fear learning by acting in peripheral β_2_-adrenoceptors. In this study (Alves et al., 2016), we used a fear conditioning test which is a test to evaluate emotional learning and fear memory but does not induce an anxiety disorder. On the other hand, now we report the induction of PTSD in mice (Pynoos et al., 1996; Pawlyk et al., 2005; Louvart et al., 2006; Bali and Jaggi, 2015), an anxiety disorder, with a more intense traumatic stimulus (days 0 and 1) followed by exposure to repeated situational reminders (days 2 and 7), which induces an increase in the anxiety response (at least until day 10). In this PTSD mice model, we observed significantly strong positive correlations between contextual freezing on day 7 and EPI in the adrenal gland, plasma, and urine.

We show that the release of EPI may be important for the formation and persistence of traumatic memories in PTSD since freezing behavior in EPI-deficient mice (Pnmt-KO) after re-exposition to the context was lower than in WT mice in the PTSD-induction groups. Moreover, EPI peripheral administration restored traumatic contextual memory in EPI-deficient mice (Pnmt-KO) on day 7 of PTSD induction, which suggests a causal role for EPI. Therefore, peripheral EPI appears to strengthen contextual traumatic memories in PTSD. EPI or vehicle was given on days 0, 1, 2, and 7 since EPI has a short half-life (3- to 5-min; Khan, 2006). It remains unanswered if the pre-treatment with EPI only on days 0 and 1 (traumatic stimulus) is sufficient to influence behavior on day 7 in Pnmt-KO mice. On the other hand, we have previously reported that Pnmt-KO mice appear to have a decrease in β_2_-adrenoceptor vasodilating effects (Bao et al., 2007; Moreira-Rodrigues et al., 2014). In fact, at rest, Pnmt-KO mice blood pressure was shown to be a little higher than in WT animals (Mendes et al., 2016), and during stress, such as acute exercise, Pnmt-KO mice were shown to have significantly increased blood pressure (Bao et al., 2007). Therefore, we cannot exclude a possible increase in blood pressure during stress to influence behavior.

EPI is a hydrophilic hormone and thus does not cross the blood-brain barrier (Weil-Malherbe et al., 1959). On the other hand, the administration of EPI directly to the brain does not seem to be physiologic. Also, Pnmt has very little expression and enzymatic activity in the brain (Ho et al., 1974; Lew et al., 1977; Mefford, 1987), however, we cannot exclude that epinephrine neurons may ascend to the hippocampus. One of the most common hypotheses is that glucose may mediate EPI’s action in the central nervous system (Morris et al., 2013). We have already suggested that after a fear event, EPI is released from the adrenal gland to the bloodstream. Afterward, EPI may act in β_2_-adrenoceptors inducing liver cells to release a transient glucose surge into the blood which may be a mediator in the central nervous system strengthening contextual fear memories (Alves et al., 2016; Oliveira et al., 2018).

The elevated plus-maze is described as a method for assessing anxiety-like responses of rodents (Pellow et al., 1985). This test is based on the natural tendency of animals to avoid open and elevated places, as well as on their natural exploratory behavior in novel environments (Zhang et al., 2012). Also, in the light–dark transition test, the time spent in the light is a good index of anxiety-like behavior (anxiogenic activity; Takao and Miyakawa, 2006). We performed behavioral tests consisting of the elevated plus-maze, light–dark transition, and open field with a 24-h interval between each test. Our findings are consistent with previous results suggesting that WT mice in the PTSD-induction group exhibit an anxiogenic-like behavior (Li et al., 2006; Zhang et al., 2012) since it was shown an elevated freezing response associated with a decrease in the number of open arms entries and time spent in the light compartment in these mice. A significantly strong negative correlation was observed between contextual freezing on day 7 and open arms entries in WT mice in the PTSD-induction group. Crawley (1981) and Crawley et al. (1984) have previously described an increased number of transitions between compartments as a key index of anxiolytic action. Thus, EPI-deficient Pnmt-KO mice seem to present lower anxiety levels than WT mice in the PTSD-induction groups, since they showed a decrease in freezing behavior and an increase in the number of open arms entries and transitions between compartments.

Furthermore, in the open field test, no significant differences were observed in the total distance traveled, number of squares crossed, and entries in the center, and in all groups, no correlations were observed between contextual freezing on day 7 and open-field parameters. These results are following a previous study which showed that foot shocks associated with situational reminders did not affect the locomotor activity of male mice in an open field test performed 3–6 weeks after the first foot shock of the PTSD mice model (Pynoos et al., 1996). Thus, this seems to support the idea that the aversive procedure did not affect the animals’ spontaneous locomotor activity (Li et al., 2006; Zhang et al., 2012). Also, Toth et al. (2013) have previously shown that Pnmt-KO mice did not show any alteration in spontaneous locomotor activity compared to WT mice, which is in agreement with our results.

Taking into consideration the contextual traumatic memory that arises from PTSD induction, we decided to investigate the hippocampal gene transcription to further understand the molecular basis of this pathology. It is known that the hippocampus is a brain structure involved in the formation of contextual fear memories and also that to form long-term memories there is a requirement for new gene transcription and protein synthesis (Flexner et al., 1963; Phillips and LeDoux, 1992). *Nr4a* genes are considered to be immediate early genes because their expression can be rapidly induced by different stimuli (Morgan and Curran, 1989). Previous studies showed *Nr4a* subfamily genes expression in the hippocampus to be upregulated after training of fear conditioning procedure contributing to contextual memory consolidation on the next day (von Hertzen and Giese, 2005; Hawk and Abel, 2011; Hawk et al., 2012). Also, we have already shown that glucose may be a mediator of EPI in the central nervous system and could be important to induce the expression of the *Nr4a* family genes involved in contextual fear memories (Oliveira et al., 2018). The NR4A transcription factors induce the expression of several target genes, such as the brain-derived neurotrophic factor (*Bdnf*) gene suggested to play an important role in hippocampus-dependent contextual fear memory (Mizuno et al., 2012). Another gene target is proto-oncogene c-Rel which seems to be necessary for learning and consolidation of hippocampal-dependent memory (Ahn et al., 2008).

Three hours after day 0 of PTSD model induction we show an increase in *Nr4a2* and *Nr4a3* mRNA expression in the hippocampus of WT mice in the PTSD-induction group in comparison to control. Since WT mice in the PTSD-induction group exhibited higher freezing response after contextual exposure, these results suggest that *Nr4a2* and *Nr4a3* hippocampal mRNA expression might be involved in the consolidation of traumatic memories in PTSD on day 2. Moreover, *Nr4a1*, *Nr4a2*, and *Nr4a3* mRNA expression was not altered in the Pnmt-KO mice PTSD-induction group in comparison to control and this may contribute to lower contextual traumatic memory observed in these mice 2 days after PTSD induction. Since *Nr4a* subfamily genes are immediate early genes, they are more prone to change their expression only a few hours after the stimulus and afterward return to baseline levels (Morgan and Curran, 1989; von Hertzen and Giese, 2005; Hawk and Abel, 2011; Hawk et al., 2012). However, it remains unanswered if exposure to repeated situational reminders (days 2 and 7) is a sufficient stimulus to increase *Nr4a* subfamily genes expression again on day 2 and day 7. On day 7 of the PTSD induction model, WT mice maintained a high freezing response after contextual exposure, contrary to Pnmt-KO mice. Therefore, EPI seems to enhance the persistence of traumatic memories which may contribute to the dysfunction of these memories’ extinction. This is in agreement with what is observed in patients with PTSD which show deficits in the extinction of traumatic memories (Lissek et al., 2005; Inslicht et al., 2013).

In conclusion, the decrease of freezing behavior presented by mice lacking endogenous EPI (Pnmt-KO mice) supports the idea that EPI may be required to the formation and persistence of contextual traumatic memories in PTSD through enhancement of the expression of *Nr4a2* and *Nr4a3* genes in the hippocampus. Peripheral administration of EPI restored traumatic memories in Pnmt-KO mice, which suggests a causal role for EPI. The persistence of contextual traumatic memories may contribute to anxiety-like behavior and resistance of traumatic memory extinction in this PTSD mice model.

## Data Availability Statement

The raw data supporting the conclusions of this article will be made available by the authors, without undue reservation.

## Ethics Statement

The animal study was reviewed and approved by Organism Responsible for Animal Welfare in Faculty of Medicine of University of Porto and National Authority for Animal Health.

## Author Contributions

MM-R and RM conceived the study. RM performed most of the experiences and respective statistical analysis. AO, GC, MM, RS, and PS performed some experiences and respective statistical analysis. RM and MM-R reviewed the statistical analysis, interpreted results, and wrote the manuscript. All authors contributed to the article and approved the submitted version.

## Conflict of Interest

The authors declare that the research was conducted in the absence of any commercial or financial relationships that could be construed as a potential conflict of interest.
