# Supplementary material for: Epinephrine May Contribute to the Persistence of Traumatic Memories in a Post-traumatic Stress Disorder Animal Model
Source: Front Mol Neurosci. 2020 Oct 26;13:588802. doi: 10.3389/fnmol.2020.588802 (PMC7649334; doi:10.3389/fnmol.2020.588802)
Supplement: Supplementary file 1 [file Data_Sheet_1.pdf]

Supplementary Material

Supplementary Data

1. Statistic details of results

1.1 EPI increases after PTSD induction

Regarding parameters measured during metabolic studies, Two-Way ANOVA repeated measures showed a main effect of time in food intake [F (2, 16) = 39.8, $p < 0.0001$, $\eta_p^2 = 0.83$, Figure 2A] and in 24-hour urine volume [F (2, 21) = 12.1, $p = 0.0003$, $\eta_p^2 = 0.54$, Figure 2D].

1.2 EPI-deficient mice have less freezing after PTSD induction than WT

On days 0 and 1 of PTSD induction model, Two-Way ANOVA revealed a main effect of pathology in vocalization [F (1, 27) = 906.3, $p < 0.0001$, $\eta_p^2 = 0.97$, Figure 4A; F (1, 27) = 118.4, $p < 0.0001$, $\eta_p^2 = 0.97$, Figure 4B] and jump [F (1, 27) = 8.1, $p = 0.0085$, $\eta_p^2 = 0.22$, Figure 4A; F (1, 27) = 23.1, $p < 0.0001$, $\eta_p^2 = 0.46$, Figure 4B] responses.

On days 0 and 1, Three-Way ANOVA repeated measures showed a main effect of time [F (6, 150) = 99.5, $p < 0.0001$, $\eta_p^2 = 0.79$, Figure 4C; F (6, 150) = 6.6, $p < 0.0001$, $\eta_p^2 = 0.21$, Figure 4D] and pathology [F (1, 25) = 858.9, $p < 0.0001$, $\eta_p^2 = 0.97$, Figure 4C; F (1, 25) = 364.7, $p < 0.0001$, $\eta_p^2 = 0.93$, Figure 4D], and a time x pathology interaction [F (6, 150) = 99.5, $p < 0.0001$, $\eta_p^2 = 0.79$, Figure 4C; F (6, 150) = 6.6, $p < 0.0001$, $\eta_p^2 = 0.21$, Figure 4D].

Furthermore, Three-Way ANOVA repeated measures showed a main effect of pathology [F (1, 21) = 327.5, $p < 0.0001$, $\eta_p^2 = 0.94$, Figure 4E; F (1, 20) = 80.1, $p < 0.0001$, $\eta_p^2 = 0.81$, Figure 4F], genotype [F (1,21) = 3.93, $p = 0.0407$, $\eta_p^2 = 0.16$, Figure 4E; F (1,20) = 9.1, $p = 0.007$, $\eta_p^2 = 0.31$, Figure 4F], and a pathology × genotype interaction [F (1,21) = 3.6, $p = 0.043$, $\eta_p^2 = 0.14$, Figure 4E; F (1,20) = 8.7, $p = 0.008$, $\eta_p^2 = 0.31$, Figure 4F] in freezing behavior on days 2 and 7.

1.3 EPI-deficient mice have less anxiety after PTSD induction than WT

In the elevated plus maze test 8 days after PTSD induction, Two-Way ANOVA revealed a main effect of pathology [F (1, 48) = 6.6, $p = 0.0136$, $\eta_p^2 = 0.12$, Figure 5A; F (1, 48) = 4.5, $p = 0.0386$, $\eta_p^2 = 0.086$, Figure 5B] in open arms entries and in the total number of arm entries, and a genotype × pathology interaction [F (1, 48) = 11.1, $p = 0.0016$, $\eta_p^2 = 0.19$, Figure 5A] in open arms entries.

In the light-dark transition test 9 days after PTSD induction, Two-Way ANOVA indicated a main effect of pathology [F (1, 52) = 15.6, $p = 0.0002$, $\eta_p^2 = 0.23$, Figure 6B; F (1, 48) = 15.4, $p = 0.0003$, $\eta_p^2 = 0.24$, Figure 6C] in the time spent in the light and in the dark compartment, a main effect of genotype [F (1, 48) = 6.6, $p = 0.0136$, $\eta_p^2 = 0.12$, Figure 6D] in the total numbers of transitions, and a genotype × pathology interaction [F (1, 48) = 5.63, $p = 0.0217$, $\eta_p^2 = 0.11$, Figure 6A] in light-dark latency time.

1.4 Peripheral EPI may be involved in the persistence of traumatic memories in PTSD

In freezing behavior, Two-Way ANOVA repeated measures showed a main effect of time [F (4, 48) = 10.9, $p < 0.0001$, $\eta_p^2 = 0.47$, Figure 8E; F (4, 48) = 3.8, $p = 0.0095$, $\eta_p^2 = 0.24$, Figure 8F] on days 2 and 7, a drug effect [F (1, 12) = 33.9, $p < 0.0001$, $\eta_p^2 = 0.74$, Figure 8F] on day 7, and a time × drug interaction [F (4, 48) = 2.7, $p = 0.0405$, $\eta_p^2 = 0.18$, Figure 8E; F (4, 48) = 3.7, $p = 0.0099$, $\eta_p^2 = 0.23$, Figure 8F] on days 2 and 7 of PTSD induction model.

1.5 EPI appears to contribute for the persistence of traumatic memories in PTSD by influencing Nr4a genes expression in the hippocampus

Two-Way ANOVA showed a genotype × pathology interaction [F (1, 46) = 6.4, $p = 0.0149$, $\eta_p^2 = 0.12$, Figure 9B; F (1, 46) = 11.7, $p = 0.0013$, $\eta_p^2 = 0.20$, Figure 9C] in mRNA expression of *Nr4a2* and *Nr4a3* genes.
